# The landscape of metastatic progression patterns across major human cancers

**DOI:** 10.18632/oncotarget.2677

**Published:** 2014-11-04

**Authors:** Jan Budczies, Moritz von Winterfeld, Frederick Klauschen, Michael Bockmayr, Jochen K. Lennerz, Carsten Denkert, Thomas Wolf, Arne Warth, Manfred Dietel, Ioannis Anagnostopoulos, Wilko Weichert, Daniel Wittschieber, Albrecht Stenzinger

**Affiliations:** ^1^ Institute of Pathology, Charité University Hospital, Berlin, Germany; ^2^ German Cancer Research Center (DKFZ), Heidelberg, Germany; ^3^ Massachusetts General Hospital/Harvard Medical School, Department of Pathology, Boston, MA, USA; ^4^ Institute of Pathology, University Hospital Heidelberg, Heidelberg, Germany; ^5^ Institute of Legal Medicine, University Hospital Münster, Münster, Germany; ^6^ German Consortium for Translational Cancer Research (DKTK), Heidelberg, Germany; ^7^ National Center for Tumor Diseases (NCT), Heidelberg, Germany

**Keywords:** autopsy, metastasis, systemic disease, carcinoma, solid tumor, cancer, survival

## Abstract

The majority of patients with solid malignancies die from metastatic burden. However, our current understanding of the mechanisms and resulting patterns of dissemination is limited. Here, we analyzed patterns of metastatic progression across 16 major cancer types in a cohort of 1008 patients with metastatic cancer autopsied between 2000 and 2013 to assess cancer specific progression patterns of disease and related risk predictions. The frequency and location of metastases were evaluated in and across 1) 16 major cancers, 2) smoking- and non-smoking-related cancers and 3) adeno- and squamous cell carcinoma. Associations between primary and secondary sites were analyzed by the fractional and the relative risk methods. We detected significantly different cancer specific patterns of metastatic progression with specific relative risk profiles for secondary site involvement. Histology and smoking etiology influenced these patterns. Backward analysis showed that metastatic patterns help to predict unknown primary sites. Solid malignancies maintain a unique and recurrent organ tropism to specific secondary sites which does not appear to be strongly influenced by advances in cancer medicine as shown by comparison with previous data sets. The delineated landscape of metastatic progression patterns is a comprehensive data resource to both clinical and basic scientists which aids fostering new hypotheses for cancer research and cancer therapies.

## INTRODUCTION

In solid malignancies, not the primary tumor but metastatic spread and systemic disease account for approximately 90% of cancer-related deaths [1]. Both, the clinical and pathological staging system of tumors strongly rely on this observation and specify nodal and distant spread of the primary tumor with increasingly dismal prognosis thus serving as a rationale to stratify patients into different treatment arms according to their stage of disease [2]. Over the years, great advances in our understanding of malignant transformation from healthy tissue via initiation, promotion, and progression towards the primary tumor were made [3]; however, the precise biological mechanisms by which individual malignant tumors actually metastasize to specific secondary sites still remain largely unexplored [4]. Our current conceptualized knowledge of systemic tumor expansion highlights two main routes through which metastases may occur and influence metastatic patterns, i.e., i) the microenvironment of the ‘host’ organ and ii) the vasculature structure and blood flow [5-8]. Among others [9-11], additional key players contributing to tumor spread are the exceedingly complex and highly dynamic spatial and temporal evolution of tumor clones during disease progression and therapy [12] and circulating metastasis-initiating cells potentially derived thereof [13, 14].

So far, several variably powered clinical and pathological studies have reported on the specific distribution and frequency of metastases of various malignancies [15-25]. These studies have substantially improved our comprehension of the biology and clinical behavior of individual tumors and not only aid in daily clinical reasoning and decision making but also foster the generation of scientific hypotheses and pave the way for new fields of research [26]. Until now, autopsy is the gold standard to definitively determine total metastatic disease burden and the current TNM classification even restricts assignment of certain pM stages to post-mortem examinations [2]. Notably, while most work has focused on single tumor entities to our knowledge there are only two studies which have investigated metastatic patterns across malignancies from different anatomical sites in large autopsy series reflecting the broad spectrum of tumors encountered in a clinical setting. Of these, the landmark study on carcinomas by Abrams et al. [27] dates back more than 60 years and a more recently published well-powered study by diSibio and French [28] employing a similar approach is built upon a dataset from 1914 to 1943. Both studies do not reflect current cancer medicine since treatment strategies potentially influencing tumor development and progression have considerably evolved over the last century. Two other studies of which one was restricted to adenocarcinoma [29] and the other one aimed at predicting patterns of metastases across cancers linked to disease progression [30] retrieved clinical but no definitive autopsy data from the beginning of the 1990s for their respective analyses.

Hence, we set out to comprehensively analyze the spatial distribution and frequency of metastases in i) 16 major types of solid tumors ii) two more coarse-grained but clinically highly relevant tumor classes, i.e., smoking- vs. non-smoking-related cancers and adenocarcinomas vs. squamous cell carcinomas in a recent (2000-2013) cohort of 1008 metastatic cancer patients autopsied at the Charité University Hospital. The objectives of the study are a) to identify common metastatic progression patterns within and across different solid tumor types and b) to identify metastatic patterns which help to infer the primary site of the tumor in clinical routine diagnostics.

## RESULTS

### Cohort characteristics and overview of the study design

**Figure 1:**
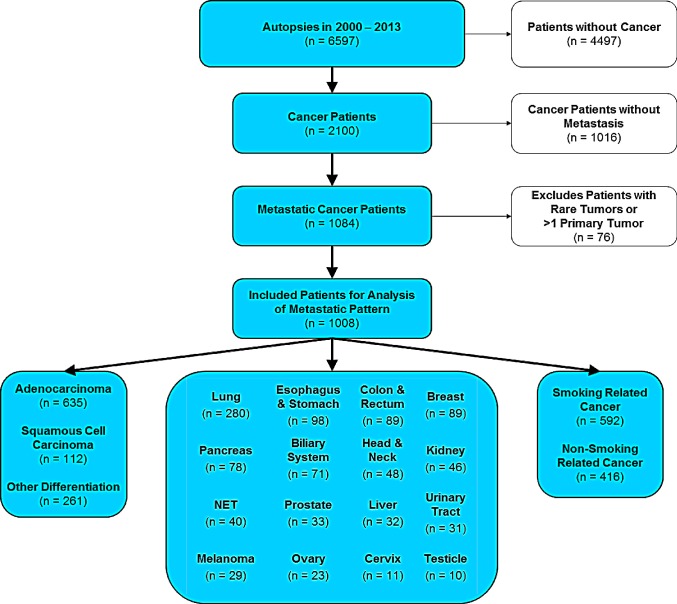
CONSORT statement (flow diagram) Out of 6597 patients autopsied in the years 2000-2013 at Charité hospital, 1008 patients were included in the study. Inclusion criterion was a diagnosed metastatic cancer disease with primary location at one of the 16 most common primary sites. Cancer diseases originating from more than one primary site were excluded from the study.

Between 2000 and 2013, a total number of 6597 patients were autopsied at the Charité Institute of Pathology. For further statistical analysis, cases without cancer (n=4497) as well as cancer cases without metastases (n=1016) were excluded. Due to low prevalence, single cases of rare tumors or metastatic cases with more than one primary were not incorporated leaving a total cohort of 1008 autopsied patients with metastatic solid malignancies (Figure 1). 57% of the patients were male, 43% of the patients were female, median age at autopsy was 64. For statistical analysis, tumors were classified into 1) 16 major cancer types, 2) adenocarcinoma vs. squamous cell carcinoma and 3) smoking- vs. non-smoking-related tumors as described previously [31]. Additionally, 20 different metastatic sites were recorded as stated in the Patients and Methods section.

### Frequency of metastases across 16 major cancer types and to 20 secondary sites

**Figure 2:**
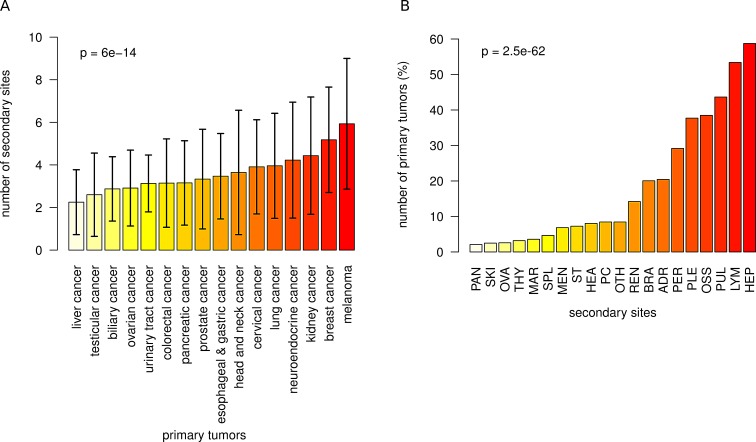
Frequency of metastases across 16 major cancer types A) Number of metastasis sites (mean and sd) for each of the primary sites. The mean number of metastasis sites increased from 2.3 (liver cancer) via 4.0 (lung cancer) to 5.9 (melanoma). Different primary tumors had a significantly different number of metastasis sites (p=6.0E-14). B) Percentage of primary tumors that metastasized to 20 secondary sites. The frequency of metastatic hits strongly depends on the localization of the secondary site (p=2.5E-62).

First, we evaluated whether the 16 major tumor entities were associated with different frequencies of metastases across the anatomical sites recorded. As displayed in Figure 2A, we detected a gradual increase of the number of metastases with melanoma and breast cancer (mean 5.9 and 5.2, respectively) as tumors with a high frequency of metastases at the one end of the spectrum and liver cancer with a comparably low number of metastasis (mean 2.3) at the other end of the spectrum. Differences in the frequency of metastases across primaries were highly significant (p=6.0E-14). Second, we calculated the number of affected patients separately for each secondary site (Figure 2B). We observed strong differences in the number of metastatic hits to secondary sites (p=2.5E-62), where liver, non-regional lymph nodes, lung, bones and pleura were affected frequently (59%, 53%, 44%, 38% and 38%) while pancreas, skin, ovary, thyroid, bone marrow and spleen showed low numbers of metastases (all frequencies <5%).

### Distribution and patterns of metastatic spread across 16 major cancer types

**Figure 3:**
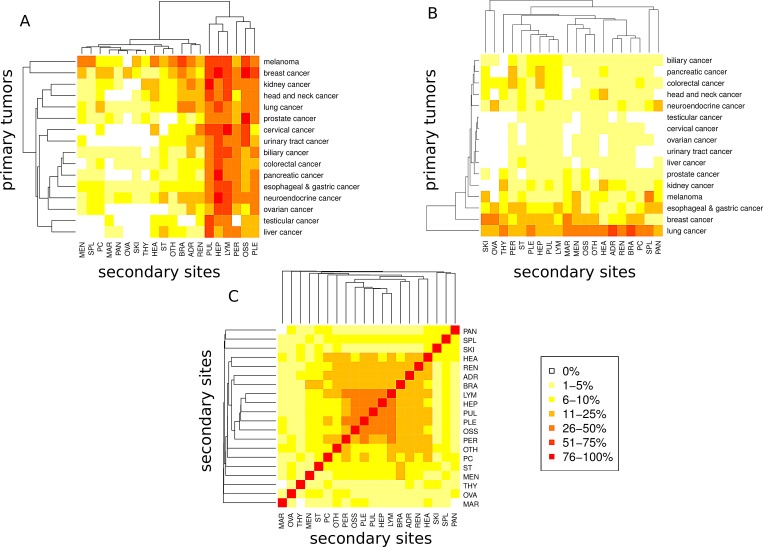
Metastatic progression from 16 primary sites to 20 secondary sites A) Percentage of primary tumors progressing to a secondary site. B) Percentage of metastases originating from a primary site. C Co-occurrence analysis of metastases. For each pair of secondary sites, the percentage of primaries with metastases at both sites relative to the number of primaries with metastases minimum at one of the two sites is shown.

Next, we analyzed metastatic patterns for all major cancer types and used heatmaps for intuitive visualization of metastatic clusters. Figure 3A demonstrates how often a given primary cancer in our cohort spreads to one of the 20 different anatomical sites. For example, while breast cancers tended to metastasize to liver, bones, non-regional lymph nodes, lung and pleura (80%, 79%, 60%, 54% and 52%), prostate cancers were predominantly associated with metastatic spread to the bone (91%) and much lower frequencies (<50%) of metastases in other anatomical sites. Figure 2B illustrates a reverse scenario, which infers the quantitative contribution of cancer types to the metastatic burden of a secondary site. For example, lung and breast cancer accounted for the majority (24% and 7%) of brain metastases while splenic metastasis frequently occurred from lung cancers and melanomas (32% and 26%). As depicted in Figure 2C, we also found patterns of co-occurrence among metastases across cancers. For example, pulmonary metastases were often associated with metastatic spread to distal lymph nodes, liver, pleura and the bones (61%, 60%, 49% and 47%).

### Relative risk analysis of metastasis across 16 major cancer types

**Figure 4:**
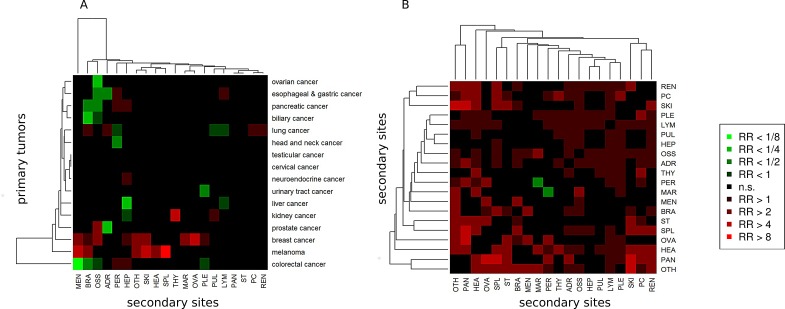
Relative risk (RR) analysis of 16 primary and 20 secondary sites A) RR for progression from a primary site to a secondary site. B) RR for co-occurrence of two secondary sites. Significantly enhanced RRs (red boxes) and significantly reduced RRs (green boxes) compared to the entire cohort.

Complementing our results on metastatic patterns and to narrow in on the clinical situation, we additionally calculated the relative risk (RR) for each major cancer to metastasize to one of the 20 recorded distant anatomical sites as shown in Figure 4A. Calculation of RRs and significance assessment allowed to identify associations that are enriched or depleted compared to the entire cohort of 1008 primary tumors. Melanoma showed strongly enhanced metastatic progression to spleen (RR=8.9), skin (RR=5.6) and meninges (RR=4.0). Breast cancer showed strongly enhanced metastatic progression to the ovaries (RR=4.8). Moreover, in Figure 4B, we plotted the relative risk with which two metastases from different anatomical sites co-occur. The strongest enrichment of co-associations between two metastatic sites was detected between pancreas and skin, pancreas and ovaries as well as pancreas and spleen (all RRs >5).

### Patterns of metastatic progression

**Figure 5:**
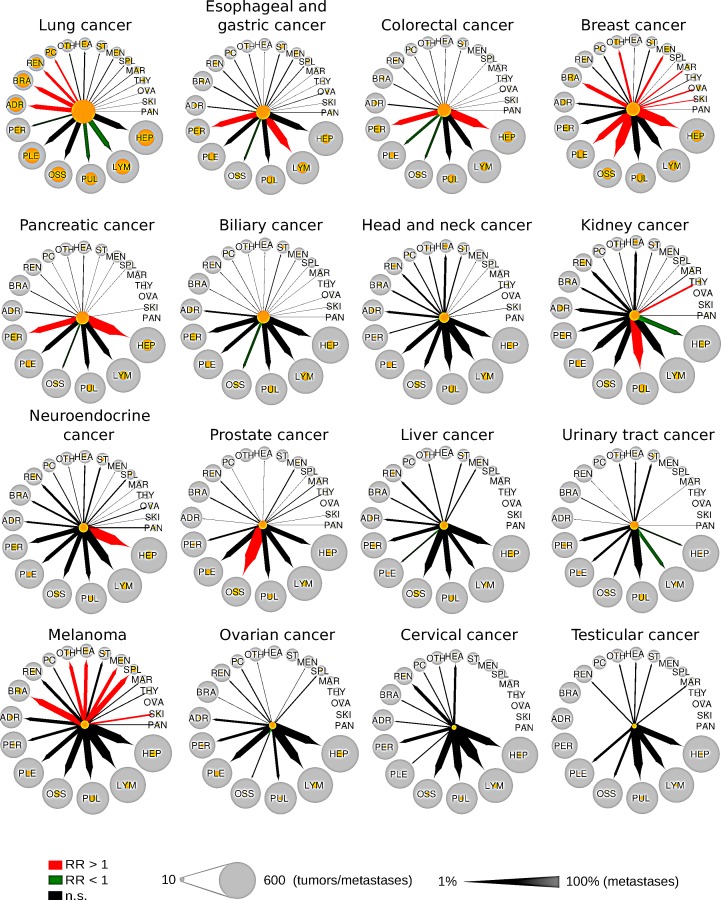
Metastatic progression from 16 primary sites to 20 secondary sites Circle size is proportional to the number of tumors or metastases. Orange circles refer to the number of metastases from a single primary site. Arrows width is proportional to the percentage of tumors that metastasize from a primary site to a secondary site. Colored arrows refer to significant enrichment (red arrows) or a significant depletion (green arrows) of a metastatic route. RR = relative risk.

Figure 5 summarizes the data from our pattern and risk analysis as clock plots for every major cancer entity. For example, while the RR of breast cancer to metastasize to the liver, bone and pleura was enhanced (RR=1.4, 2.0 and 1.4), the RR of bone metastases in pancreatic cancer was diminished (RR=0.37), but enhanced for liver metastases and peritoneal spread (RR=1.4 and 1.7). In contrast, melanomas showed an increased RR for spreading to specific anatomical sites (including spleen, meninges, brain and heart) which are usually not at high risk when compared to metastatic patterns of other major cancers.

### Influence of smoking and histology on metastatic patterns

**Figure 6:**
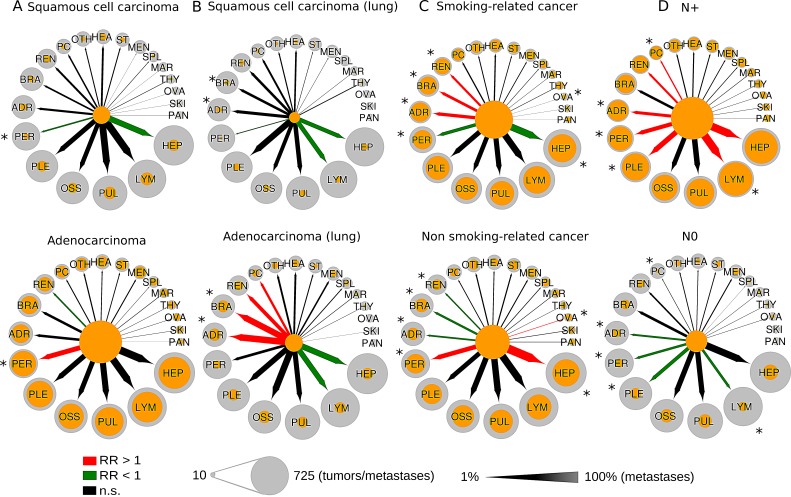
Comparison of the metastatic progression in selected tumor subgroups A) squamous cell cancer vs. adenocarcinoma, B) squamous cell lung cancer vs. adenocarcinoma of the lung, C) smoking-related vs. non-smoking-related cancer, D) node-positive vs. node-negative cancer. Circle size is proportional to the number of tumors or metastases. Orange circles refer to the number of metastases from a single primary site. Arrows width is proportional to the percentage of tumors that metastasize from a primary site to a secondary site. Colored arrows refer to significant enrichment (red arrows) or a significant depletion (green arrows) of a metastatic route. RR = relative risk, * = significant different metastasis frequency between subgroups.

We also tested whether spread to secondary sites is influenced by exposure to tobacco smoke and the phenotype of tumor cells. To this end, we classified all cancers investigated here in smoking- and non-smoking-related cancers and separately stratified cancers in either squamous cell carcinoma or adenocarcinoma where applicable (for details see Patients and Methods section as well as Figure 1). As depicted in Figure 6, we found significant differences in the metastatic patterns of squamous cell carcinoma and adenocarcinoma, smoking- and non-smoking-related cancers as well as for node-positive and node-negative cancer. When applying the histological classification to all primary sites, we detected significantly increased numbers of metastases to the peritoneum in adenocarcinoma compared to squamous cell carcinoma (36% vs. 11%, p=2.2E-18). In a subgroup analysis of lung cancer, we found adenocarcinoma compared to squamous cell carcinoma metastasizing significantly more often to the brain (46% vs. 20%, p=0.0021) and to the adrenal gland (49% vs. 24%, p=0.0045). In smoking-related cancer compared to non-smoking-related cancer, we found significantly increased numbers of metastases to the adrenal gland (26% vs. 13%, p=7.4E-07), to the kidney (17% vs. 10%, p=0.00047) and to the brain (23% vs. 16%, p=0.0065). Furthermore, we found node-positive (N+) cancers to have a drastically increased probability to spread to distant lymph nodes compared to node-negative (N-) metastatic cancer (63% vs. 19%, p=4.2E-25). The association between metastases in local lymph nodes and metastases in distal lymph nodes could be found in many of the cancer types including biliary cancer, breast cancer, colorectal cancer, gastric cancer, liver cancer, lung cancer, neuroendocrine cancer and ovarian cancer. Furthermore, the probability to metastasize to peritoneum (33% vs. 17%), to pleura (42% vs. 26%), to adrenal gland (24% vs. 13%) and to pericardium (10% vs. 4%) was significantly increased in N+ cancer compared to N-cancer. In contrast, the percentage of bone metastases (38% vs. 39%), brain metastases (20% vs. 20%) and lung metastases (43% vs. 39%) did not change much. Finally, sex was not significantly associated with distinct patterns of metastases (data not shown).

### Prediction of primary cancers by metastatic patterns

**Figure 7:**
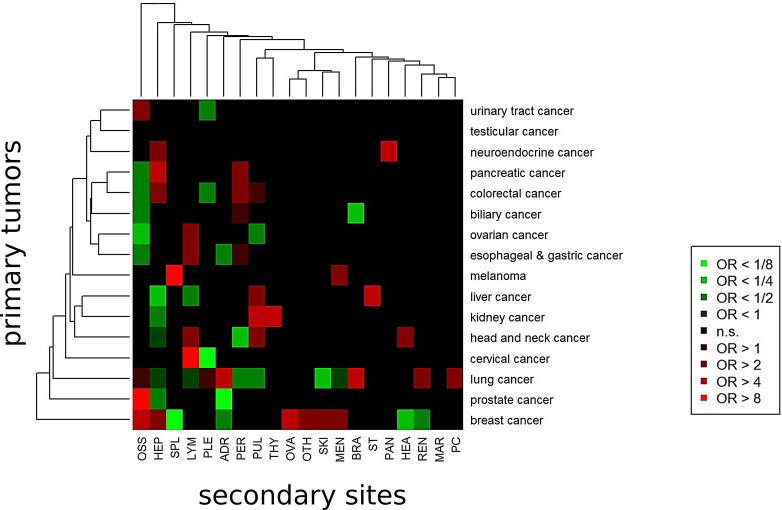
Models for prediction of the primary cancer sites from secondary cancer sites (metastases) For each primary site, multivariate logistic regression was executed to obtain odd ratios (ORs) associated with the secondary sites. Secondary sites associated with significantly higher probability for the primary site (red boxes) and secondary sites associated with significantly lower probability for the primary site (green boxes).

Given the frequent clinical question of an unknown primary in the setting of widely metastatic disease, we investigated whether distant metastasis location may be used to predict the primary tumor thereby reflecting a clinical scenario where the primary localization has to be infered in patients presenting with metastatic disease. As shown in Figure 7, metastases in the pericardium, kidney, brain and adrenal glands, for instance, were associated with a high probability to originate from lung cancer (OR=3.03, OR=2.73, OR=4.21 and OR=4.54), while the probability that skin metastases stem from lung cancer was low (OR=0.23). Bone metastases had a high probability to stem from a primary of the prostate or breast (OR=25.64 and OR=6.78), but had a low probability to occur from primaries of the gastrointestinal system or of the ovaries (OR=0.13, OR=0.31 and OR=0.45). As yet another example, breast cancer was characterized by a fairly high probability of metastatic spread to the bone, liver, ovaries, skin and meninges (OR=6.78, OR=2.94, OR=7.80, OR=3.80 and OR=3.05) while the chance of distant metastases in heart and kidney was low (OR=0.23 and OR=0.33). Thereby, our data provide an up-to-date dataset to support determination of the site of origin in the cancer of unknown primary in a clinical setting.

Using multinomial regression, we investigated the degree, how much the metastasis patterns were predictive for the type of primary tumor. To this end, we used a multiple random sampling approach, where 1000 training sets of 672 patients (2/3) were randomly drawn and the remaining patients (1/3) were included in the test sets. Using this approach, 34% (29% - 38%) of primary locations were predicted correctly, for 48% (44% - 53%) of patients the true primary location was among the two top predictions and for 59% (54% - 63%) of the patients the true primary location was among the top three locations. Using the approach of the top three locations, more than half of the primary locations could be predicted for lung cancer (91%), breast cancer (81%), pancreatic cancer (64%), prostate cancer (61%), colorectal cancer (59%) and esophageal and gastric cancer (56%).

### Lifetime analysis

Finally, we investigated the influence of the location of the primary site and the localizations of secondary sites on the total cancer-related lifetime of the patients. It should be noted that this kind of analysis is different from an analysis of clinical outcome, where not the total lifetime, but the remaining lifetime after cancer diagnosis or surgery is analyzed. The lifetime analysis describes the burden of tumors and metastases at defined primary and secondary sites for a population rather than the prognosis for an individual patient after cancer diagnosis. Metastases in the ovaries (HR=3.4, p<0.001), the heart (HR=1.6, p=0.001), the brain (HR=1.5, p<0.001), the meninges (HR=1.4, p=0.022), the distal lymph nodes (HR=1.2, p=0.009) and the bones (HR=1.2, p=0.34) were associated with a significantly shortened lifetime. Moreover, compared to the other metastatic cancers, we found a significantly prolonged lifetime for metastatic prostate cancer (HR=0.45, p=0.001) and a significantly shortened lifetime for metastatic testicular cancer (HR=32.8, p<0.001) and metastatic esophageal/gastric cancer (HR=1.3, p=0.043). The former result corresponds to generically late occurrence and slow progress of prostate cancer compared to other cancer types, the later result reflects that testicular cancer often occurs early during lifetime.

## DISCUSSION

While solid malignancies confined to the primary site are potentially amenable to therapy with curative intent, the majority of these tumors will either primarily present as extended disease with spread to distant anatomical sites or evolve towards systemic disease over time. Despite current advances, it is the metastatic spread where even sophisticated novel approaches of multimodal therapy will eventually fail. It is therefore not surprising that metastatic cancer accounts for more than 90% of cancer-related deaths [1, 6]. Impacting both clinical work and basic research, we still struggle with understanding why and how specific tumors metastasize to specific anatomical sites with different propensities.

Here, we provide a comprehensive analysis of metastatic patterns across major cancer types that is built upon definitive autopsy data rather than clinical data potentially entailing problems with the inherently limited sensitivity and more importantly specificity of clinical and radiographic examinations. Our work provides a detailed up-to-date account of differences in metastatic progression across solid malignancies in a cohort comprising the last 14 years (2000-2013) complementing and extending previous approaches [26, 27] but also serving as a current framework to foster research efforts in both basic science and clinical oncology. Moreover, following up on the elegant study by Chen and colleagues [30] we aimed at defining and predicting networks of metastatic progression, which may help to infer the primary tumor localization when patients present with metastatic disease or so called (with dash) cancer of unknown primary (CUP) [37].

We observed strongly (and significantly) different metastasis patterns across 16 major cancer types. Very interestingly, two earlier autopsy studies by Abrams et al. [27] and diSibio and French [28] employed a similar approach and therefore in principle allow for a comparison between different eras of cancer medicine (1914-1947 vs. 2000-2013). Comparing the number of patients, our study included almost the same number as Abrams et al. [27] did 65 years ago. Both collectives comprise cases from a relatively limited period of five and 13 years, respectively. In contrast, the much larger number of cases presented by diSibio [28] mirrors a significantly longer time period of thirty years. A brief overview of the study characteristics is provided in Supplementary Table 1. However, In this context, it is important to note however, that none of the three studies was designed for direct cross-comparison and the composition of the respective patient collectives including risk factors and frequencies of cancer types among others certainly vary to some extent. Nevertheless, cautious comparison may yield some insight since diSibio and French specifically stated in their paper [28] that the patients included in their study neither received chemo- nor radiotherapy while the patient analyzed in our cohort were treated in accordance with the German guidelines effective at the time which includes neoadjuvant as well as adjuvant chemo- and radiotherapy regimens.

Interestingly, our work and the study by diSibio and French [28] reflecting the years 1914-1943 yielded similar results with breast, lung and kidney cancer developing high numbers of metastases and, for example, liver cancer with a comparably low metastases frequency. Moreover, DiSibio and French [28] reported the most frequently affected secondary sites as regional and distant nodes followed by liver, lung and bone; a rank order which is in line with our data (not accounting for regional lymph nodes) albeit at somewhat different frequencies (see Supplementary Table 1). Abrams et al. [27] provided detailed data on several cancer types, including breast and lung cancer with lung, bone, pleura and liver as the most frequently affected secondary sites in breast cancer patients and lung, liver, adrenal glands and bone as the most frequent metastatic sites in lung cancer patients,respectively. These results collected in the 1940s are in good accordance with our data set compiled at the beginning of the 21^st^ century. In summary, these data might indicate that despite considerable improvements in therapy over the last century metastatic progression patterns are quite robust and do not seem to be strongly influenced by advances in cancer medicine.

**Figure 8:**
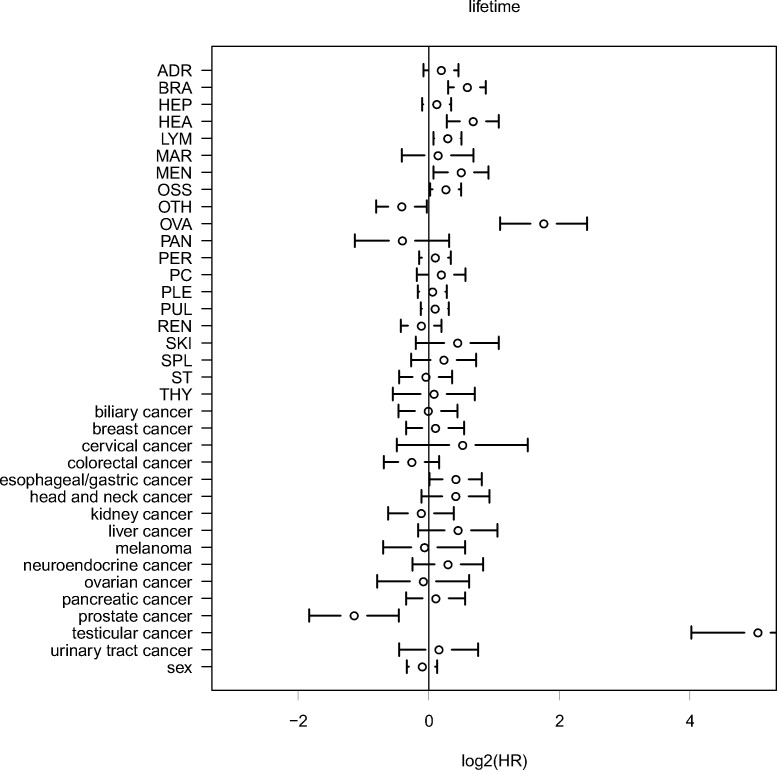
Multivariate analysis of lifetime using Cox proportional hazard modeling Analysis of the influence of the primary tumor site and of secondary tumor sites on total lifetime. Different than in overall survival analysis (time of survival after the first cancer diagnosis) the total lifetime (patient age at death) is analyzed. Only cancer-related deaths are taken into consideration. HR = hazard ratio.

Given the fact that, for example, melanomas have a higher rate of metastatic spread compared to each of the other major cancer our data also suggest that each tumor has a distinct and specific capacity and efficiency to evolve cell clones which are capable of spreading and seeding in a given period of time. These results match current findings demonstrating cancer-specific intratumoral heterogeneity [12] eventually fostering cancer-specific evolution of metastatic clones [10] as already conceptualized by Nowell in 1976 [38].

Moreover, our results clearly demonstrate that apart from tumor-specific metastasis rates cancers also have entity-specific predilection sites for tumor expansion. As already hypothesized by Ewing in 1919 [39], these are certainly -in part-attributable to the anatomical structure of vessels and blood flow as the underlying transport vehicle and prerequisite for hematogenous spread or the lymphatic system with the lymphatic vessels as possible entry and exit point for the tumor cells. Starting with clinical observations of contiguous development of metastases in axillary lymph nodes of breast cancer patients reported by Halsted in 1907 [40], nowadays it is well established that lymph node metastases are a prognostic factor for organ metastases in melanoma and particularly breast cancer [41,42]. However, the stepwise progression of malignant cells from tumor to sentinel lymph nodes and from thereon to distal lymph nodes and organs has not been demonstrated conclusively [43]. Our results showing that node-positive cancers of different entities (including breast, colorectal and gastric cancer among others) spread much more often to distant lymph nodes than node-negative cancers (63% vs. 19%, p=4.2E-25, Figure 6D) suggest that cellular mechanisms facilitating access to the local lymphatic system and hence the lymphatic system are crucially required for metastatic dissemination to distant lymph nodes and challenge the view that spreading from local to distant nodes is primarily enabled by blood vessels. Moreover, as shown by us, several node-positive cancers also appear to be have a higher likelihood to spread to distant sites compared to node-negative cancers. These findings may support the notion that the lymphatic system in conjunction or even interaction with blood vessels contributes to the colonization of distant sites.

However, anatomy and physiology of the hematologic and lymphatic alone hardly explain why, according to our study, e.g., melanomas are particularly prone to metastasize to the spleen or prostate cancer as well as breast cancers are bone-seeking malignancies whereas colorectal cancers are not. In this context, it is noteworthy that our results also demonstrate a variable risk of co-occurrence of two metastases at different anatomical sites with some pairs (e.g. ovarian and pancreatic metastases) at high risk and others (e.g. bone marrow and peritoneum) at low risk. Originally proposed as ‘seed and soil’ hypothesis by Paget in 1889 [44], these results clearly imply mechanisms beyond anatomy where either certain molecular features acquired during tumor evolution serve as one master key for distinct locks or the microenvironment of two host organs resemble each other thereby equally facilitating metastasis in two anatomically distinct locations.

As illustrated and summarized in Figure 5, every single cancer type investigated here shows a specific organ tropism resulting in recurrently affected secondary sites, which characterizes every tumor biologically and in turn clinically. Matching the data from Chen et al. [30] we detected e.g. for colorectal cancer statistically significant increased relative risks to spread to liver and peritoneum, which according to the findings from Chen et al. [30] is already inherent at the time of diagnosis and obviously conserved during tumor evolution until death. In contrast, melanomas display a peculiar pattern of relations to various secondary sites whereas kidney cancer shows a strong risk for pulmonary metastases followed by spread to bones, pleura and peritoneum but a comparably lower risk to metastasize to the liver.

We also show that the progression patterns from primary to secondary sites is influenced by tumor tissue histology and etiology of smoking. As the tumor phenotype is an integral expression of genomic, epigenetic, proteomic and other biological characteristics in cancer cells, it is likely that phenotypic characteristics also reflect specific metastatic patterns. Among others [45], smoking directly damages the genetic integrity of cells, specifically by G·C to T·A transversions and hence smoking history can be traced by specific genetic signatures imprinted throughout the cancer cell genome [46]. With these considerations, differences in metastatic behavior between smoking- and non-smoking-related cancers do not appear unlikely.

Our findings indicate that every primary tumor appears to harbor the capability to facilitate a specific pattern of distant colonization which is dynamic over the course of disease but highly recurrent across individual cancer of the same entity. Hence, it might provide new insights to shift the view on solid tumors away from the sole primary confined to a certain organ eventually prone to metastasize towards a disease where tumor cells of origin establish and maintain a network to specific secondary sites with ties of different strength. Of note, this view does not challenge the stochastic foundations of metastatic progression which play a crucial and well established role but acknowledges the notion that besides randomness there are obviously nonrandom determinants [47] that ultimately result in or -in other words-can be readout as recurrent and cancer type specific patterns of metastatic disease. This network may be maintained by cancer specific circulating tumor cells trafficking along pre-established anatomical routes (links) which acquired specific properties in the primary by chance that in principle allow colonization of distant organs [14,48] and also self-seeding [49] of the primary tumor (nodes). While the overall success rate of metastatic spread appears to be rather low [47] and is influenced by randomness it is striking that once colonization is successful, a recurrent pattern of affected organs emerges across individual tumors of the same entity. As shown by us and others [27,28], there is a certain degree of overlap between metastatic patterns of distinct entities which may be influenced by anatomical conditions but may also suggest common traits of the respective primaries. It is also tempting to speculate whether organ-specific colonization is influenced by -possibly developmentally-imprinted molecular similarities shared between the organ from which the primary evolves and secondary sites colonized by the primary. Comparing our results with the data from diSibio and French [28], Abrams et al. [27] and Chen et al. [30] as well as considering reduced cancer mortality over the last 100 years [50], the occurrence of cancer-specific network-like spreading to distant sites may be delayed or even halted in some cases but seems not to be prevented or fully disrupted by current systemic therapies despite significant advances in the understanding of cancer and in cancer medicine.

In summary, the delineated landscape of metastatic progression provides a comprehensive resource for clinical oncologists, physician-scientists and basic scientists. Our current comprehensive analysis of metastatic patterns across major cancer types in a large autopsy series strongly supports the assumption by Polzer and Klein [13] as well as other groups that our efforts in understanding when and how specific progression networks evolve and being maintained rather than exclusively focus on the primary tumor crucially require significant enhancement by inclusion of the possible pathways of metastatic progression to develop innovative and effective strategies abrogating metastatic disease in the future.

## PATIENTS AND METHODS

### Study cohort and ethical approval

The retrospective study using anonymized patient data was approved by the local ethics committee (application number: EA1/077/14). All adult autopsy reports performed at the Charité Institute of Pathology (Campus Charité Mitte and Campus Virchow Hospital), Berlin, Germany, from the years 2000-2013 were reviewed retrospectively. Out of a total number of 6597 autopsied patients, 1008 patients diagnosed with metastatic cancer progressed from one of 16 primary cancer sites were included in the study (Figure 1). Patients with hematological malignancies and sarcomas were excluded. All patients underwent a complete clinical autopsy, including histological assessment of all major internal organ systems including the brain. For each patient, a definitive structured autopsy report stating final anatomical diagnoses was established that included detailed gross and microscopic findings as well as clinicopathologic correlations including case discussion. Autopsies were performed according to established protocols and reviewed and presented by at least one board-certified pathologist as described previously [31, 32]. Briefly, each autopsy followed a standard operating procedure including macroscopic and microscopic examination, the former comprising external inspection, initial dissection and internal examination. Evisceration was usually achieved by a modified en bloc method of Ghon which preserves organ blocks (brain, cervico-thoracic, upper abdominal part, small and large intestine, genitourinary) and thus enables evaluation of the interrelationship of organs, lymphatic drainage and vasculature (accounting for regional disease in an organ system). In single cases organs were removed one by one. This was followed by three dimensional inspection of each organ and organ system. Hollow organs, ducts and vessels were opened to evaluate the inner linings and surfaces. Organs were sliced serially at approx. 1.0 cm intervals in parasagittal, coronal and horizontal planes as appropriate. For oncological cases, microscopic sampling included any lesion or tissue alteration (lower limit: approx. 0.3 cm in diameter) that appeared suspect at macroscopic evaluation. Generally, there were no restrictions on the extent of examination. Additionally, standard sampling (in the absence of evident macroscopic alterations) included the heart including coronary arteries, left and right lung, liver, pancreas, spleen, and bone. For each case, all collected tissue samples were formalin fixed and paraffin embedded (FFPE) with osseous tissue decalcified in EDTA prior to FFPE. Subsequent microscopic evaluation with bright field microscopes was primarily performed on HE-stained slides, accompanied by additional histochemical stains and immunohistochemistry if necessary.

### Cancer classification

For statistical analyses, we recorded age, sex, life span, the cause of death, tumor type, number of metastases and metastatic sites. The total cohort was subdivided into different tumor types as depicted in Figure 1.

Three classification schemes were used for a specific analysis of metastatic patterns across cancers. First, primary tumors were stratified into 16 anatomical sites (lung, esophagus and stomach, colon and rectum, breast, pancreas, biliary system, head and neck, kidney, neuroendocrine tumors, prostate, liver, urinary tract, melanoma, ovary, cervix and testicles). Secondly, patients were classified in two main histological tumor types: adenocarcinoma (n=635) and squamous cell carcinoma (n=112). Third, tumors were grouped according to the major risk factor smoking. To this end, the degree of relation to tobacco smoking was graded based on the *population attributable fraction* (PAF) as reported previously [32,33]. Specifically, tumor types with PAF ≥20% were classified as smoking-related, and cancer types with PAF <20% were classified as non-smoking-related. As a result, smoking-related cancers (n=592) comprised lung, head and neck, esophagus and stomach, kidney, pancreas, urinary system and cervix cancer. Non-smoking-related cancers (n=416) included malignancies of the colon, rectum, liver, ovary, breast, biliary tract, prostate, testicle as well as melanoma and neuroendocrine cancer.

In addition to full UICC staging [2], we employed a more fine-grained classification scheme that accounts for the most common metastatic sites throughout the body. The anatomical sites of metastatic spread of each metastasis were recorded as follows: adrenal gland (ADR), bone (OSS), bone marrow (MAR), brain (BRA), heart (HEA), kidney (REN), liver (HEP), lung (PUL), non-regional lymph nodes (LYM), meninges (MEN), ovary (OVA), pancreas (PAN), pericardium (PC), peritoneum (PER), pleura (PLE), skin (SKI), soft tissue (ST), spleen (SPL) and thyroid (THY). Metastases occurring at rarer sites were recorded as other site (OTH); resulting in a total of 20 different locations. Lesions in the bone marrow which were ambiguous at the macroscopic level but confirmed as metastasis by microscopy were classified as MAR while osseous metastases already apparent on the macroscopic level with involvement of cortical and cancellous bone and corroborated by histology were classified as OSS.

### Data analysis

The software environment R [34] was used for statistical computing and graphic production. Values of p<0.05 were considered statistically significant. The number of metastatic sites (mean and sd) was calculated for each of the primary sites and significance was assessed using the Kruskal-Wallis rank test.

Two kinds of analysis methods were applied to analyze the association between primary and secondary sites or between two secondary sites: The *fractional method* simply estimates the fraction of tumors that metastasize from a primary to a secondary site, while the *relative risk (RR) method* quantifies enrichment or depletion of an association compared to the situation in the entire cohort. For two localizations *a* and *b* (either both secondary sites or *a* primary and *b* secondary site) the RR was calculated as

$$RR(a,b)=\frac{f(\text{site }a\text{ and site }b\text{)}}{f(\text{site }a)f(\text{site }b)},$$

wherein the expressions *f*(…) denote relative frequencies with respect to the total number of patients in the study. Significances of RRs were assessed using Fisher's exact test.

Hierarchical clustering of percentages was conducted using the Manhattan distance as dissimilarity measure and average linkage to compare between clusters. For relative risk (RR) and odds ratio (OR) analysis, log2 data were used as input for heatmap and clustering analyses. Hierarchical clustering of RRs and ORs was conducted using the Euclidean distance as dissimilarity measure and average linkage to compare between clusters.

To identify significantly enriched or depleted targets of metastatic progression, p-values were corrected using the Benjamini-Hochberg method (FDR control at 5%) separately for Figures 5, 6A, 6B, 6C and 6D. Metastatic progression patterns were visualized using Cytoscape [35] and the R package RCytoscape [36]. Node size was chosen proportional to the number of tumors or metastases. Edge thickness was chosen proportional to the percentage of primary tumors that metastasize to a secondary site.

Two approaches were employed to infer the primary cancer location from the secondary locations: First, separately for each of the 16 primary locations, we executed multivariate logistic regression with the 20 secondary locations as covariates. As results, we obtained odd ratios (ORs) describing an increased or decreased probability for a tumor at a primary site associated with a metastasis that occurred at one of the secondary sites. Second, as in Chen et al. [30], a model for prediction of the primary site was fitted using multinomial logistic regression. The logistic model was validated using a multiple random cross-validation protocol: 1000 training sets of 672 patients (2/3) were randomly drawn and the remaining patients (1/3) were included in the test sets. Prediction success was reported as mean value with 95% confidence intervals estimated from the 1000 training-test splits.

Analysis of cancer-related lifetime was executed using Cox proportional hazard modeling in SPSS (IBM Corporation) with sex, primary tumor sites and secondary metastasis sites as covariates. Hazard ratios (HRs) of the primary sites were calculated relative to lung cancer that was the most common primary site. Only cancer-related deaths were considered as event, cases with other causes of death were included in the analysis, but marked as censored. Significance of the covariates was assessed using the Wald test.

## SUPPLEMENTARY MATERIAL TABLE


