# Supplementary material for: The landscape of metastatic progression patterns across major human cancers
Source: Oncotarget. 2014 Nov 4;6(1):570–83. doi: 10.18632/oncotarget.2677 (PMC4381616; doi:10.18632/oncotarget.2677)
Supplement: Supplementary file 1 [file oncotarget-06-570-s001.pdf]

The landscape of metastatic progression patterns across major human cancers

Supplementary Material

Supplementary Table 1: Overview of three different autopsy studies investigating metastatic disease progression across major cancer types.

	**Autopsy studies on metastatic progression patterns across major human cancers**		
General study characteristics	**Abrams et al. (1950)**	**diSibio/French (2008)**	**Budczies et al. (2014)**
Major aims of the study	- description of metastatic patterns	- description of metastatic patterns	- description of metastatic patterns<br>- determination of the frequency of metastases<br>- prediction of primary sites
No. of cases analyzed	1000	3827	1008
Period in which autopsies were performed	1943 – 1947	1914 – 1943	2000 – 2013
Tumor origin	epithelial	epithelial	epithelial
Treatment	n/a[#]	No chemotherapy or radiation	Surgery, chemotherapy, and radiation
No. of primary cancers analyzed	18* (36)	41	16
No. of metastasis sites analyzed	44	30	20
Additional influencing factors investigated	-	-	- tobacco smoking<br>- histological type
Basic and comparable results of the analyses			
The most frequent primary cancers in the cohort investigated	1. breast (16.7%)<br>2. lung** (16.0%)<br>3. stomach (11.9%)<br>4. colon (11.8%)<br>5. rectum (8.7%)	1. rectum (11.4%)<br>2. breast (11.3%)<br>3. cervix (10.9%)<br>4. stomach (9.1%)<br>5. prostate (5.0%)	1. lung (27.8%)<br>2. esophagus/stomach (9.7%)<br>3. colorectal cancer (8.8%)<br>4. breast (8.8%)<br>5. pancreas (7.7%)
The most frequent metastasis sites	1. abdom. LN (49.5%)<br>2. liver (49.4%)<br>3. lung (46.5%)	1. reg./dist. LN (20.5%/12.9%)<br>2. liver (11.1%)	1. liver (59%)<br>2. dist. LN (53%)<br>3. lung (44%)

	4. mediast. lymph nodes (42.1%)	3. lung (10.7%)<br>4. bone (6.5%)	4. bone (38%)

Therapy data not provided in the paper

* These entities account for about 95% of all cases.

** No distinction between NSCLC and SCLC

LN = lymph nodes
